# The Effect on Human Balance of Standing with Toe-Extension

**DOI:** 10.1371/journal.pone.0041539

**Published:** 2012-07-25

**Authors:** Pei Xuan Ku, Noor Azuan Abu Osman, Ashril Yusof, Wan Abu Bakar Wan Abas

**Affiliations:** 1 Department of Biomedical Engineering, Faculty of Engineering, University of Malaya, Kuala Lumpur, Malaysia; 2 Sports Centre, University of Malaya, Kuala Lumpur, Malaysia; University of South Australia, Australia

## Abstract

**Background:**

Postural balance is vital for safely carrying out many daily activities, such as locomotion. The purpose of this study was to determine how changes in normal standing (NS) and standing with toe-extension (SWT) impact postural control during quiet standing. Furthermore, the research aimed to examine the extent to which the effect of these factors differed between genders.

**Methodology/Principal Findings:**

Thirty healthy young adults (age = 21.2±1.3 y; height = 1.63±0.07 m; mass = 56.0±9.3 kg) with no prior lower limb injuries participated in the study. A postural stability test using the Biodex Balance System was used for both NS and SWT conditions. The three measurements from the BBS were Overall Stability Index (OSI), Medial-Lateral Stability Index (MLSI) and Anterior-Posterior Stability Index (APSI). No significant difference was found between NS and SWT in the OSI, MLSI or APSI (*F*_2, 28_ = 3.357, *p* = 0.077). The main difference between the stability index scores was significant (*F*_2, 28_ = 275.1, *p*<0.001). The Bonferroni post-hoc test showed significant differences between the OSI and MLSI (*p*<0.001); the OSI and APSI (*p*<0.001); and the MLSI and the APSI (*p*<0.001). Significant differences were found during NS (*p*<0.001), for the MLSI when compared with the APSI, but this was not found during the SWT condition. Additionally, no gender effects were proven to exist that altered postural sway during quiet standing.

**Conclusions/Significance:**

This study reveals significant interaction between the stability indices measured; OSI, APSI and MLSI in both NS and SWT. Standing with toe extended does not have a significant impact on an individual’s ability to control their balance during normal quiet standing. However, the findings revealed that the sway tendency in the medial-lateral direction might serve as a factor in an individual’s ability to regain balance.

## Introduction

Since balance is a vital prerequisite component of life for all human beings, balance control has been examined extensively in a number of studies. All movements that are performed under static or dynamic conditions are necessary to maintaining a good quality of life and hence it is important that individuals can achieve body equilibrium and maintain stability during quiet standing and ambulatory activities.

Quiet standing is a complex task that involves the integration of multiple body segments, joints and sensory systems in order to regulate balance while attempting to stay upright in a static position without moving. Current statistics show that there are approximately 13,042,000 fatal falls throughout the population of the United states in year 2010 and a lack of balance control against perturbation is believed to be one of the biggest contributors to such accident [1].

In general, the somatosensory, vestibular and visual systems are believed to be the key physiological inputs of balance control [2], [3]. Researchers agree that the human body will attempt to orientate the center of mass (CoM), which is a virtual point that is equivalent to the total body mass at which the average of mass distribution for each body segment may be assumed to be concentrated, against perturbation [4]. A healthy human being will naturally attempt to return to the CoM within the base of support by subconsciously regulating the body’s position. The base of support is the displacement region for the center of pressure (CoP), since the CoP serves as the location point of the average distribution for all the pressure over the ground surface contact area [5]. By measuring the displacement of the CoP, an individual’s balance can be assessed. Winter [6] argued that the location of the CoP is directly impacted by an individual’s foot activity. He stated that the CoP will move in a medial direction as a result of an increase of evertor activity, while the CoM shifts laterally, and will move in a lateral direction as a result of an increase in the invertor activity, which results in the medial shift of the CoM [7].

Numerous studies have investigated the important role that the function of the foot plays in standing; heel-toe standing, heel-standing and full toe-standing [8]. One of the most important factors in achieving postural balance is the anatomy of the foot. The contribution of the foot mechanism towards the stability of postural standing has attracted attention from various investigators. In particular, it has been proven that the normal arch in the foot plays an important role in shock absorption and propulsion phases, whereas the toe is responsible for providing a stable surface area that remains in contact with the ground and serves to relay relevant sensory proprioception information to the central nervous system. This information is then used to perform basic corrections when balance disturbances occur [9]. Cavanagh et al. [10] reported that 60% of weight-bearing pressure is distributed at the heel of the foot, 8% at the mid foot, and 28% at the forefoot. With regards to pressure distribution, studies have found that the highest peak pressure at the toes were found at the great toe (30%), followed by the second toe (24%), third toe (21%), fourth toe (16%) and small toe (9%) [11]. In addition, the second and third metatarsal areas display the highest pressure at the forefoot region for a normal, healthy individual [10], [12]. According to Hicks [13], the extension of toes may subsequently lead to the following situations: (i) the elevation of the foot arch, (ii) the supination of the feet occurring at the posterior part of the foot, (iii) a lateral rotation of the leg, (iv) a tightness of the band at the region of the plantar aponeurosis.

In recent studies, the Biodex Balance System SD (BBS; Biodex Medical Systems Inc., Shirley, NY, USA) has been more frequently used as a tool for balance assessment than the formally-popular force platform. The BBS is a multiaxial device that evaluates and measures the CoP for up to 20° of platform tilt in a 360° range of motion. BSS can compute three measures: the Overall Stability Index (OSI), Medial-Lateral Stability Index (MLSI) and Anterior-Posterior Stability Index (APSI). These variables were used in this study to measure and evaluate balance performance. The purpose of this study was to determine how changes in normal standing (NS) and standing with toe-extension (SWT) conditions impact postural control. Furthermore, the study sought to examine the extent to which these impacts differ according to gender. The researchers hypothesized that the standing postures of NS and SWT would not impact the postural sway in terms of the OSI, APSI or MLSI.

## Materials and Methods

### Ethics Statement

The study protocol was reviewed and approved by the Medical Ethic Committee in University of Malaya Medical Centre. All participants gave their written informed consent to participate in the study.

### Participants

Thirty healthy young adults (15 male and 15 female) with no prior lower-limb injuries were recruited for this study. None of the participants had experienced previous balance training using BBS, or had suffered from any neurological, vestibular or balance impairments. The research incorporated a crossover study design, where all participants underwent the same test protocols. In order to certify the health condition of the participants with regards to their daily routines and lifestyle, a basic lifestyle survey was undertaken before the study began. Participants who engaged in an active lifestyle and who generally wore flat shoes were selected for this study.

### Instrumentation

The BBS used in this study contained four strain gauges under a circular platform in order to measure the displacement of CoP at a sampling rate of 20 Hz. For the OSI, MLSI and APSI stability scores, these indices were the measures of standard deviation, which were assessed along the path of sway around the zero point from the center of the platform of BBS. The units were recorded in degrees. The foot displacements that occurred on the medial-lateral (ML) axis were labeled as “x-direction”, while those on the anterior-posterior (AP) axis were labeled as “y-direction,” and these variables were measured as the MLSI and APSI respectively. In turn, the OSI, which is sensitive to the change in body sway across the ML and AP axes, was derived from the MLSI and APSI. The formulas for OSI, MLSI and APSI are shown below:
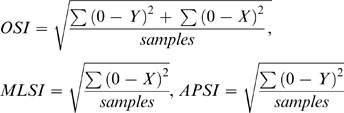



Arnold and Schmitz [14] suggested that the OSI is an important tool that can be used to assess balance stability, since the OSI can be affected by both AP and ML directions. Previous studies have found that the reliability of the BBS for 5 trials were 0.92 (OSI), 0.90 (MLSI) and 0.86 (APSI). Within this study, Biodex Medical Systems software (Version 1.33) was used to compute the transmitted data and generate the stability indices that were used to evaluate the data.

### Study Protocol

**Figure 1:**
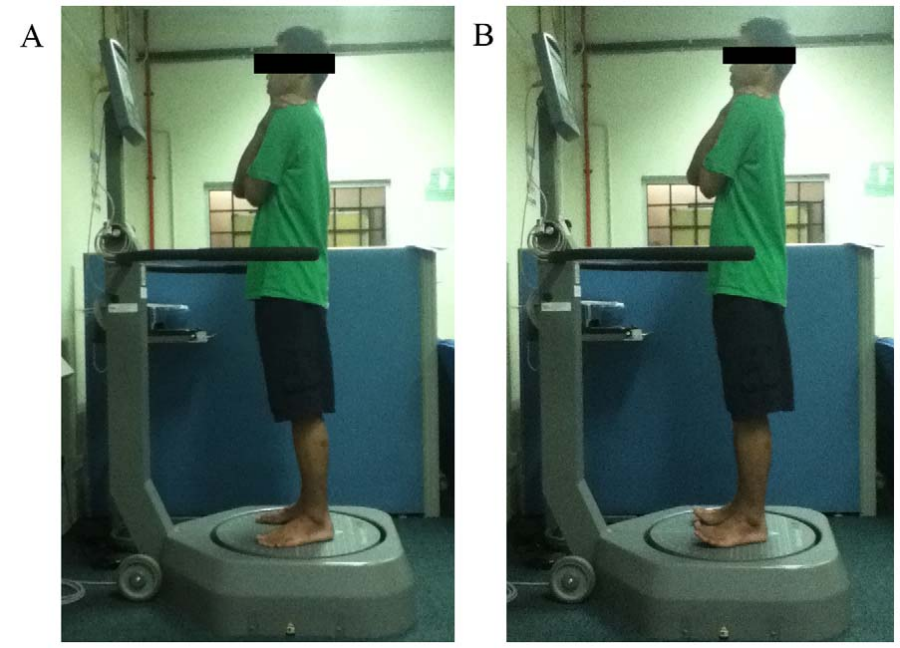
Experimental setup (**A**). Illustration for the setup that allow the balance assessment during normal standing by using the Biodex Balance System SD. (**B**). Illustration for the assessment during the standing with toe-extension condition.

**Figure 2:**
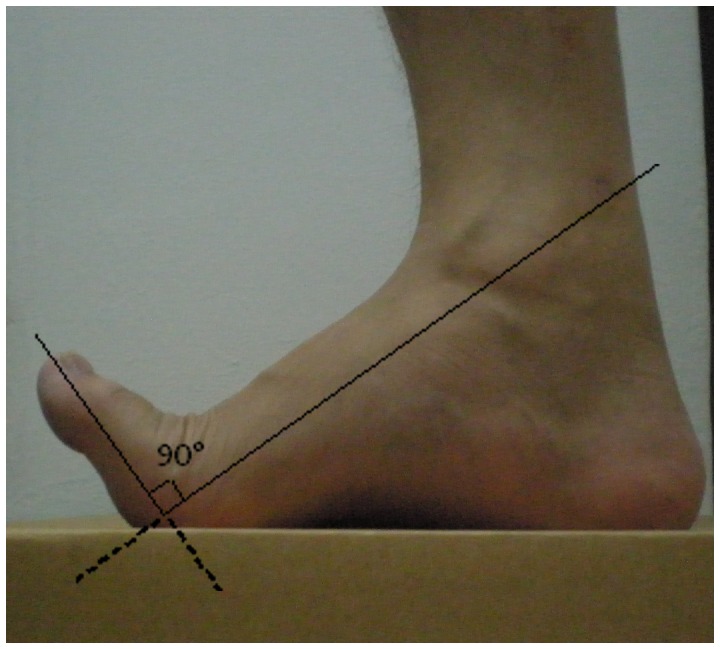
Foot posture for standing with toe-extension. Illustration for the balance assessment of standing with toe-extension condition using BBS.

In the current study, the BBS was used to evaluate the static postural control during (i) normal standing (NS) (Figure 1A), and (ii) standing with toe-extension (SWT) (Figure 1B). For the static level, the circular platform was set to remain static with no platform tilt allowed. The participants randomly performed the balance tests for both sets of tests. During NS, participants were instructed to stand barefooted on the BBS platform. Both of their hands were placed over their chests and they were asked to look straight forward. They were then instructed to adjust both their supporting feet in order to achieve a comfortable standing posture that allowed them to maintain balance. The instructions for the position adjustment were provided on the instrument panel. Once the participant was in a comfortable standing position, the platform was locked and the foot placement of the participant subsequently remained constant throughout the test. When the test commenced, participants were required to concentrate on maintaining the moving pointer at the center of the circle on the instrument panel for a period of 30 s. Each participant underwent 5 trials and a rest period of 10 s was provided between each trial to enable the participant to momentarily relax their body without looking at the panel. Five trials were performed continuously and the average score was computed. The same procedure was applied during the SWT as that utilized in NS test, excluding the element that involved the standing posture. During the SWT, participants were instructed to lift their toes as high as they could (normally about 90° to the line of metatarsal as shown in Figure 2) throughout the test in order to avoid ground contact [13]. Real-time foot monitoring was employed to monitor the angle of the participant’s toes. A verbal reminder was given if their toes dropped below the 5° limit. If the participant’s toes dropped more than 6°, the trial was cancelled and the test was repeated.

### Statistical Analyses

All statistical analysis was performed using SPSS for Windows (Version 19; IBM Corp., Armonk, NY). A 2×3×2 (standing posture × stability index × gender) mixed factor repeated measures analysis of variance (ANOVA) was used for the purposes of the analysis. There was no interaction of gender and stability index so the groups were collapsed for gender. A 2-way interaction was seen for standing posture and stability index. The Shapiro-Wilk Test was used to assess the normality of the data. The test of normality verified that all the data produced was normally distributed. The data was normalized with respect to body mass and relative stability was used in the data analysis in order to avoid a potential misinterpretation of data and to reduce the absolute stability differences [15]. Relative stability was defined as the ratio of total stability index score over body mass, which was expressed as degree per kilogram.

## Results

The descriptive statistical analysis on the demographic characteristics for all participants is tabulated in Table 1. The results of this study elucidated that there was no significant main effect between NS and SWT in the OSI, APSI and MLSI scores among the 30 participants (*F*_2, 28_ = 3.357, *p* = 0.077). The main effect of balance was significant (*F*_2, 28_ = 275.1, *p*<0.001). The Bonferroni post hoc test showed significant differences between the OSI with MLSI (*p*<0.001), the OSI with APSI (*p*<0.001) and the MLSI with APSI (*p*<0.001). The standing posture × stability index interaction was significant (*F*_2, 28_ = 13.64, *p*<0.001).

**Table 1:** Descriptive and demographic characteristic for all participants.

	Male	Female	Overall
**No. of individual (n)**	15	15	30
**Age (year)**	21.2±1.6	21.1±1.0	21.2±1.3
**Height** a **(m)**	1.67±0.06	1.59±0.06	1.63±0.07
**Mass** a **(kg)**	62.0±8.4	49.9±5.5	56.0±9.3
**BMI** a **(kg/m^2^)**	22.3±3.2	19.7±2.4	21.0±3.1

a Significantly different between gender (*p*<0.05).

With the available number of participants in this study, the within condition analysis revealed significant differences between the OSI with APSI, the OSI with MLSI, and the APSI with MLSI in NS (all *p*<0.001), while differences were shown between the OSI and APSI, and the OSI and MLSI in SWT (*p*<0.001). The mean of the relative stability index for both NS and SWT conditions are displayed in Table 2.

**Table 2:** Descriptive statistics (mean ± standard deviation) for the balance stability mean score between genders.

	Male			Female			Overall		
	Mean ± SD	95% CI	CV (%)	Mean ± SD	95% CI	CV (%)	Mean ± SD	95% CI	CV (%)
**Normal standing**									
**OSI (°)**	0.006±0.001	0.006–0.007	22.9	0.008±0.003	0.006–0.009	32.3	0.007±0.002	0.006–0.008	30.8
**MLSI (**°**)**	0.002±0.001	0.002–0.003	40.5	0.003±0.001^c^	0.002–0.004	47.1	0.001±0.002	0.002–0.003	45.7
**APSI (**°**)**	0.005±0.002^b^	0.004–0.006	35.2	0.006±0.003^b^	0.005–0.007	43.0	0.006±0.002^b^	0.005–0.006	39.8
**Standing with toe-extension**									
**OSI (°)**	0.007±0.003	0.006–0.009	40.0	0.008±0.003	0.007–0.010	36.3	0.008±0.003	0.007–0.009	37.8
**MLSI (**°**)**	0.005±0.003^a^	0.003–0.006	58.2	0.005±0.002^a^	0.003–0.006	55.6	0.003±0.005^a^	0.004–0.006	56.0
**APSI (**°**)**	0.005±0.002	0.004–0.006	38.4	0.005±0.002	0.004–0.007	35.9	0.005±0.002	0.004–0.006	37.1

a *p*<0.05: significant difference in comparison to normal standing and standing with toe-extension.

b *p*<0.05: significant difference in comparison to Medial-Lateral Stability Index (MLSI) and Anterior-Posterior Stability Index (APSI) among the same postural condition.

c *p*<0.05: significant difference between gender group among the same postural condition.

Females displayed a greater sway than males across all the conditions, although no significant differences was observed between the OSI, MLSI and APSI scores. When comparing the data of NS and SWT conditions within the male group (N = 15), significant differences were found between the OSI with APSI, the OSI with MLSI, and the APSI with MLSI (main effect *p*<0.001). Meanwhile, for the female group (N = 15), the results exhibited similar findings (main effect *p*<0.001). No differences were observed between the NS and SWT conditions between the male and female groups. Additionally, no participant grasped the holding rails to regain balance during the tests.

## Discussion

There have been numerous studies that have investigated balance stability for various standing postures. However, to date, there does not appear to be any research in existence that focuses on bilateral support standing with toe-extension. In this current study, the differences in postural stability between NS and SWT, and the impact of gender on postural control, were explored.

The results of this study substantiate the hypothesis and show that there was no difference in postural sway in NS and SWT conditions in the OSI, MLSI and APSI. The balance performance as represented by the OSI, MLSI and APSI stability scores was slightly lower during NS, but this was not significant when compared with the score achieved during SWT. Slight differences in the NS and SWT scores may be due to the fact that SWT requires more sway from the CoP as the body regains balance. According to the windlass mechanism, when the toes are extended to their limit, the plantar aponeurosis that wraps around the metatarsal bone tightens and therefore causes the arch to rise, further lifting the metatarsal head [13]. Hicks [13] claimed that the metatarso-phalangeal (MTP) head will increase the pressure of the toe against the ground and the height of the rising arch is related to the distance of the metatarsal head to the calcaneus. The current study demonstrated that the arch of the foot did rise and that the sway in both ML and AP directions insignificantly increased during as the MTP head extended. Hence, this is inconsistent with the previous findings of Hicks [13] which stated that toe-extension of the forefoot increases the postural sway due to the foot pressure that is applied at the MTP joints. As such, the toe extension may result in a reduction of the base of support and this subsequently deteriorates the stability of the body [16].

In spite of being closely matched for age (*p* = 0.892), the current study showed that gender does not influence human balance in the NS and SWT conditions, since no differences in the stability scores were found between the male and female participants. This is in contrast with previous studies, which reported that males had a greater postural sway than females during quiet standing [17]. Lee and Lin [18] suggested that the difference in body weight of males will result in greater CoP execution during the single-leg standing task. However, the findings of Mickle [17] claimed that males executed greater postural sway, even though the body weight for both genders are nearly similar.

According to the findings, the ML sway among females was slightly higher, but this was not significant when compared with that of the male control group during both the NS and SWT conditions (Table 2). Meanwhile, the current findings show that an increased complexity of standing posture leads to an increase in the ML sway. The ML sway was different than the AP sway during NS. There was a significant increase in the ML sway that was required to achieve balance, as the ML sway was similar with the AP sway in SWT. This finding may relate to the Q-angle. Studies have shown that females have a greater Q-angle than males due to the length of their femur and their bigger pelvis area [19], [20]. A larger Q-angle for females may result in an increase of rotation in hip movement, since the CoM needs to be maintained within the base of support to achieve body balance [21]. As such, greater sway will be generated in the ML direction and sideways sway will increase in response to this in order to regain body equilibrium. Maki et al. [22] also demonstrated that an increase in body sway in the ML direction might lead to an increased risk of falling. Interestingly, the findings also revealed that the value of APSI was slightly higher than MLSI across all conditions, although it showed differences in NS and no statistically significant difference in SWT. This is in agreement with previous studies which revealed that the MLSI has a low value compared to APSI [23], [24], [25]. Meanwhile, the current findings show that there is an increase in the ML sway in accordance with the increasing complexity of standing posture. The ML sway was found to be different with the AP sway during NS. There is a significant increase in the ML sway that was required to achieve balance, as the ML sway was similar to the AP sway in SWT. As such, the tendency of the ML sway was considered to be an important component for balance equilibrium when a more complex posture was applied during quiet standing.

The investigation of static balance control provides normative stability data for clinicians who are concerned with this specific toe condition. It is important to identify the changes of balance performance during SWT, since it is altered by the base of support and CoM. Some authors reported that the CoP and CoM are approximately equal only in the static or quasi-static conditions [26]. Likewise, Murray et al. [27] presented the idea that motion in the CoP is greater than that in the CoM in order to keep the CoM within the base of support, since the CoP changes in response to the CoM. From the anatomical point of view, the height of the CoM is normally lower in females than males [28]. There are also studies that investigate the effect of the type of female’s footwear on balance [29], [30]. High heel shoes tend to affect postural control by raising and shifting the CoM forward [31]. Csapo et al. [30] highlighted that the long-term use of high heels might diminish the gastrocnemius muscle fascicles and reduce the range of motion in an individual’s ankle, resulting in a feeling of discomfort even when they are wearing in flat shoes.

Although the current study was restricted to an investigation of static postural control during toe-extension standing activities, it would be interesting to compare the postural control between active toe-extension in healthy individuals and the passive toe-extension that can be caused by burn injuries. Nevertheless, the investigation of static postural control has provided a normative stability data in SWT. Besides, the sample size in this study only involved young adults (aged between 19 and 25 years old) from one specific geographical area and, as such, the data is limited.

### Conclusion

In conclusion, this study has demonstrated that differences between the NS and SWT conditions do not lead to differences in postural control. However, a small alteration in postural control during SWT shows a trend of greater amount of postural sway than NS, although this is not significantly different. The findings also revealed the interactive effect of postural control in term of the OSI, MLSI and APSI. Gender does not appear to effect static postural stability.
